# Microbial effectors target multiple steps in the salicylic acid production and signaling pathway

**DOI:** 10.3389/fpls.2015.00349

**Published:** 2015-05-19

**Authors:** Shigeyuki Tanaka, Xiaowei Han, Regine Kahmann

**Affiliations:** Department of Organismic Interactions, Max Planck Institute for Terrestrial Microbiology, Marburg, Germany

**Keywords:** virulence effector, salicylic acid, bacterial plant pathogens, fungal plant pathogens, oomycete plant pathogens

## Abstract

Microbes attempting to colonize plants are recognized through the plant immune surveillance system. This leads to a complex array of global as well as specific defense responses, which are often associated with plant cell death and subsequent arrest of the invader. The responses also entail complex changes in phytohormone signaling pathways. Among these, salicylic acid (SA) signaling is an important pathway because of its ability to trigger plant cell death. As biotrophic and hemibiotrophic pathogens need to invade living plant tissue to cause disease, they have evolved efficient strategies to downregulate SA signaling by virulence effectors, which can be proteins or secondary metabolites. Here we review the strategies prokaryotic pathogens have developed to target SA biosynthesis and signaling, and contrast this with recent insights into how plant pathogenic eukaryotic fungi and oomycetes accomplish the same goal.

## Introduction

The plant hormone salicylic acid (SA) has been extensively studied because of its influence on various plant developmental processes as well as its role on resistance to abiotic and biotic stresses (Vlot et al., 2009). In the context of biotic stress SA has been shown to be a crucial player in pathogen associated molecular pattern (PAMP)-triggered immunity (PTI) as well as effector-triggered immunity (ETI; Jones and Dangl, 2006). PAMP-triggered immunity is a plant defense reaction in which pathogens are recognized through conserved molecular patterns like flg22, an epitope of bacterial flagellin, elf18, a component of bacterial elongation factor EF-Tu, bacterial peptidoglycans, and chitin, a typical component of the fungal cell wall. PAMPs are perceived by membrane localized pattern-recognition receptors (PRRs), many of which are receptor-like kinases (RLKs) that function together with co-receptors (Macho and Zipfel, 2015). Activation of these PRRs by PAMP ligand binding elicits plant defense responses that confer a certain level of protection against virulent pathogens. PAMP-induced defense responses include calcium spiking, the production of reactive oxygen species, callose deposition which interferes with pathogen spread, the production of antimicrobial compounds, accumulation of the plant hormone SA, and the synthesis of pathogenesis-related (PR) proteins, many of which exhibit toxicity directed against the pathogen (Newman et al., 2013). Plants can also recognize an invading pathogen through secreted protein effectors and mount a highly effective defense response that is associated with programmed cell death (hypersensitive response, HR) at the site of pathogen infection. This ETI is induced by direct or indirect recognition of pathogen effectors by plant resistance (R) proteins. Direct interactions between R proteins and effector proteins have been demonstrated only rarely (Jia et al., 2000; Deslandes et al., 2003; Dodds et al., 2006). More common are indirect interactions which involve host targets that guard the R protein or act as decoy to detect pathogen effectors via R proteins, respectively (see van der Hoorn and Kamoun, 2008 for details). Pathogen effectors triggering ETI were initially identified as the products of avirulence genes (Avr). However, with the advent of whole genome sequencing and elucidation of genome-wide effectomes, Avr proteins are now included in the large group of microbial effectors and are termed effectors triggering ETI in resistant plants. R proteins typically belong to the nucleotide-binding leucine-rich repeat (NB-LRR) class, a large family of intracellular receptors (Zipfel, 2014; Macho and Zipfel, 2015), that respond to the respective pathogen effectors translocated from the pathogen to the host. Gram-negative plant pathogenic bacteria possess type III secretion systems (T3SS) to inject bacterial type III effectors (T3Es) into host cells through a specialized syringe-like structure. T3Es of pathogenic bacteria can downregulate PAMP-triggered defense responses at many levels, i.e., by direct targeting the membrane bound PRRs or their co-receptors to affect their signaling function, by specifically interfering with expression of PRR proteins, by affecting the stability of PRRs or by inactivating downstream components like MAP kinases or interfering with vesicle trafficking, which is necessary to downregulate PTI responses like callose deposition. These processes have recently been reviewed comprehensively (Macho and Zipfel, 2015) and will not be covered here as they do not specifically address SA signaling.

Eukaryotic plant pathogenic microbes like oomycetes and fungi also transfer effectors to their hosts, and this has been functionally demonstrated for many Avr proteins by expressing the respective genes in resistant hosts and demonstrating the elicitation of cell death. While this provides a simple assay for the Avr function of effectors, it is much more difficult to determine the virulence function of effectors. In addition, the mechanisms how filamentous eukaryotic plant pathogens translocate effectors are still under debate (Rafiqi et al., 2012; Doehlemann et al., 2014; Lo Presti et al., 2015).

Salicylic acid acts as a crucial signaling molecule in pathways conferring local and systemic immunity against a large number of pathogens. SA was first shown to be the key plant hormone for triggering systemic acquired resistance (SAR), an induced defense elicited by an avirulent pathogen involving the entire plant and providing protection against a broad spectrum of pathogens (Durrant and Dong, 2004). The important role of SA as a signaling molecule during basal and induced responses to virulent pathogens has been demonstrated by the isolation of plant mutants exhibiting increased susceptibility to virulent as well as avirulent pathogens. This includes the SALICYLIC ACID INDUCTION-DEFICIENT 2 (*sid2*), the ENHANCED DISEASE SUSCEPTIBILITY 5 (*eds5*), and the NON-EXPRESSOR OF PR GENES (*npr1*) mutants of *Arabidopsis thaliana*. Compared to these plant mutants NahG expressing plants, in which endogenous SA is removed by expressing a bacterial SA hydoxylase, show even stronger disease susceptibility toward virulent as well as avirulent pathogens (Cao et al., 1994; Delaney et al., 1994, 1995; Glazebrook et al., 1996; Nawrath and Metraux, 1999; Glazebrook, 2005). Furthermore it was demonstrated that SA signaling is generally important for immunity against biotrophs, while jasmonic acid (JA) and ethylene (ET) signaling confer immunity against necrotrophs (Glazebrook, 2005).

Given the importance of SA signaling in basal and induced plant defense it is clear that virulent hemibiotrophic and biotrophic pathogens that rely on living plant tissue have to downregulate SA levels to establish themselves inside the plant and cause disease. In this review we will address the intricate ways such microbes have developed to target the SA pathway to promote disease at the level of biosynthesis, signal transduction, and by affecting the crosstalk between SA and JA pathways. We will contrast modes of molecular intervention in these processes by bacterial and eukaryotic plant pathogen effectors, and highlight specifically recent findings in filamentous fungi and oomycetes.

## Effectors Interfering with SA Biosynthesis and Accumulation

**FIGURE 1:**
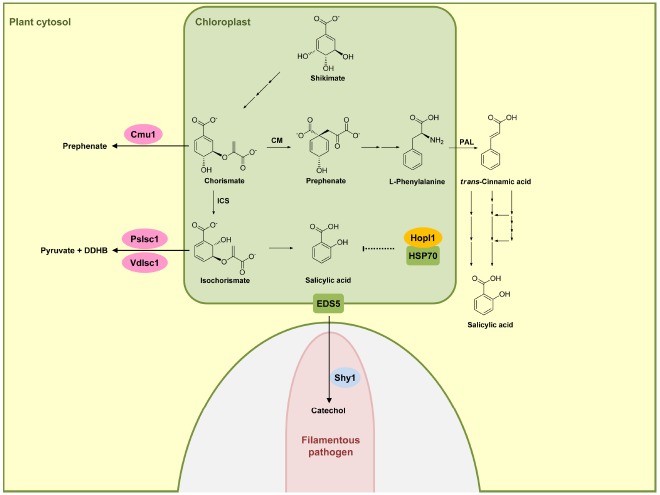
**Effectors interfering with salicylic acid biosynthetic pathway.** In plants, salicylic acid (SA) is mainly produced via the isochorismate pathway (IC) in plastids (green compartment), but can also be synthesized through phenylalanine ammonia-lyase (PAL) pathway in the cytosol. HopI1 in *P. syringae* interacts with plastidic HSP70 (green), probably affecting SA biosynthesis or transport. Secreted Cmu1 from *U. maydis* is taken up by plant cells and is proposed to rechannel chorismic acid from plastids to the cytosol, thus lowering SA levels. The oomycete *P. sojae* and the fungus *V. dahliae* secrete isochorismatases PsIsc1 and VdIsc1, respectively, converting isochorismate into 2,3-dihydro-2,3-dihydroxybenzoate (DDHB) and pyruvate. A fungal hypha is indicated in light pink in the lower part. This hypha is surrounded by the apoplast shown enlarged here (light gray) encased by the plant plasma membrane (green). Salicylate hydroxylase Shy1 (light blue) residing in the cytosol of *U. maydis*, can degrade SA. Enzymes are abbreviated: chorismate mutase (CM), isochorismate synthase (ICS). Fungal and oomycete effectors are indicated by pink ovals and bacterial effectors are indicated in dark yellow. Plant proteins are indicated by a green square. Solid arrows represent chemical reactions, dotted arrows indicate indirect inhibition and blunt ended arrows indicate inhibition (for details, see text).

In plants two distinct pathways exist for the biosynthesis of SA and both start out with chorismate, the end product of the shikimate pathway. The isochorismate pathway (IC) is operative in plastids (Figure 1). The IC pathway is the prime source for SA accumulation in non-challenged and pathogen-challenged plants (Dempsey et al., 2011; Seyfferth and Tsuda, 2014). Chorismate is converted to isochorismate by isochorismate synthase (ICS). *A. thaliana* has two *ICS* genes (*ICS1* and *ICS2*), the products of which are localized in chloroplasts. In *A. thaliana eds16* mutants and *sid2* mutants where ICS1 is defective, SA accumulation is 90% lower than in wild-type plants upon pathogen challenge (Dewdney et al., 2000). ICS2 participates only weakly in SA synthesis and its contribution is only detectable in *ics1 ics2* double-mutants (Garcion et al., 2008; Dempsey et al., 2011). Isochorismate is then converted to SA either through an isochorismate pyruvate lyase-like enzyme in the chloroplast (that has not been identified) or a chloroplast enzyme related to chorismate mutase but with a higher affinity for isochorismate (Figure 1; Dempsey et al., 2011). The transmembrane protein EDS5 from *A. thaliana* belongs to the MATE transporter family (Nawrath et al., 2002). EDS5 is chloroplast-localized (Figure 1) and presumed to play a role in exporting SA from the plastid to the cytosol (Nawrath et al., 2002; Ishihara et al., 2008). The *eds5* mutants accumulate very little SA, and display hypersusceptibility to pathogens (Nawrath and Metraux, 1999; Nawrath et al., 2002).

The second pathway for producing SA is the phenylalanine ammonia-lyase (PAL) pathway (Figure 1) in which phenylalanine is converted by PAL to *trans*-cinnamic acid, which then serves as a precursor for various routes of SA biosynthesis (Dempsey et al., 2011). Because of the minor role of this pathway in SA biosynthesis in defense signaling, we will not discuss this pathway here in detail. We will also not discuss SA modifications like glucosylation, conjugation to amino acids, or methylation (Dempsey et al., 2011), because so far these processes have not been shown to be targeted by pathogen effectors.

Turning now to effectors modulating SA biosynthesis and/or accumulation, it has been shown that the *Pseudomonas syringae* virulence effector HopI1 belongs to this group. HopI1 is targeted to plastids where it induces the remodeling of thylakoid structures. The C-terminal domain of HopI1 binds to HSP70 resulting in the formation of large complexes in association with HSP70 and the recruitment of cytosolic HSP70 to chloroplasts. It is speculated that HSP70 may be required for assembling/folding components of the SA biosynthesis or transport machinery, although a direct demonstration for this is still lacking (Figure 1). The result of HopI1 action is reduced SA accumulation (Jelenska et al., 2007, 2010).

An effector protein directly affecting SA levels is produced by the biotrophic fungus *Ustilago maydis*, which is causing smut disease in maize. Secretome analysis of apoplastic proteins from leaf tissue infected by *U. maydis* identified the secreted chorismate mutase protein Cmu1. Immunoelectron microscopy detected Cmu1 protein not only in the interface between fungal hyphae and surrounding plant plasma membrane but also in the cytosol of invaded plant cells, demonstrating that Cmu1 protein is taken up by host plant cells and functions within the cytoplasm of the plant cells. By activity assays and complementation of an *aro7* mutant of *Saccharomyces cerevisiae*, Cmu1 has been demonstrated to have chorismate mutase activity (Djamei et al., 2011). Metabolic profiling revealed that leaf tissue infected by *cmu1* mutants show increased accumulation of SA. In addition, mutants lacking *cmu1* are reduced in virulence. These results suggest that translocated Cmu1 facilitates the conversion of chorismate to prephenate to lower the availability of chorismate for SA biosynthesis (Figure 1). In this way Cmu1 is proposed to suppress SA-dependent plant defense responses, which would be harmful for a biotrophic pathogen like *U. maydis*. Upon transient expression in maize cells, Cmu1 has also been shown to spread to neighboring cells conceivably priming them for the subsequent infection (Djamei et al., 2011; Djamei and Kahmann, 2012).

A recent report demonstrated that two unrelated hemibiotrophic filamentous pathogens, the oomycete *Phytophtora sojae*, which causes root and stem rot disease in soybean, and the fungus *Verticillium dahlia*, which causes vascular wilt diseases in a large number of different plant species, secrete isochorismatases PsIsc1 and VdIsc1, respectively (Liu et al., 2014). Isochorismatases convert isochorismate into 2,3-dihydro-2,3-dihydroxybenzoate (DDHB) and pyruvate (Figure 1), thus eliminating the central precursor for SA production (Figure 1). PsIsc1 and VdIsc1 are virulence factors in both *P. sojae* and *V. dahliae* (Liu et al., 2014). Interestingly, these isochorismatases lack predicted signal sequences which direct the protein into the conventional secretory pathway. Nevertheless, these proteins are detected in culture supernatants, suggesting that they are secreted via unconventional secretion pathways. Intracellular expression of these isochorismatases in leaves of *Nicotiana benthamiana* by *Agrobacterium* infiltration significantly has been shown to reduce SA levels and increase levels of DDHB. Furthermore, transient expression of these isochorismatases in *N. benthamiana* elevates susceptibility toward the compatible hemibiotrophic pathogen *P. capsici* with a concomitant decrease in *PR-1* gene expression (Liu et al., 2014), a marker gene of the SA pathway. Thus, these filamentous pathogens attenuate SA-dependent plant defense responses by reducing the level of a crucial intermediate for SA biosynthesis (Figure 1).

Salicylic acid has been shown to be transported in the phloem (Rocher et al., 2006), and has been detected also in apoplastic fluid of *V. longisporum*-infected *A. thaliana* plants (Floerl et al., 2012). *U. maydis* has the gene for a putative salicylate hydroxylase NahG-like enzyme (*shy*1) which does not appear to be secreted (Rabe et al., 2013). Recombinant Shy1 protein indeed displays salicylate hydroxylase activity. Subsequent experiments have revealed that *U. maydis* can sense, degrade, and use SA as carbon source. However, this ability could not be linked to virulence (Rabe et al., 2013), which could either reflect redundancy or a contribution to virulence when *U. maydis* infects different plant organs. SA-degrading ability is also reported for the necrotrophic fungal pathogen *Sclerotinia sclerotiorum*, although the protein responsible for SA degradation in this organism has not yet been identified (Penn and Daniel, 2013). These studies illustrate that pathogens have developed different ways to lower SA levels in infected plants and may actually use redundant strategies to accomplish this. To what extent lower SA levels contribute to virulence appears to be variable and may depend on the system and the infection conditions.

Increased SA levels in plants depend on the expression of *ICS1* and components affecting its downstream accumulation. PAMP perception increases intracellular Ca^2+^ concentrations which regulate the activity of calmodulin (CaM) and calcium-dependent protein kinases (CDPKs). The calmodulin binding protein CBP60g positively regulates *ICS1* expression while CBP60a acts as a negative regulator (Truman et al., 2013). SAR DEFICIENT 1 (SARD1) which does not bind CaM acts redundantly with CBP60g in promoting *ICS1* transcription. Both CBP60g and SARD1 are shown to be recruited to the *ICS1* promoter region in response to pathogen attack (Zhang et al., 2010b) and consequently the induction of *ICS1* expression and SA production are significantly impaired in *sard1 cbp60g* double mutants (Zhang et al., 2010b). *ICS1* expression is furthermore positively regulated by a member of the WRKY family of transcription factors, WRKY28, whose DNA binding activity is regulated through phosphorylation (Eulgem et al., 2000; Dempsey et al., 2011; van Verk et al., 2011; Seyfferth and Tsuda, 2014). *ICS1* expression is also negatively regulated by ETHYLENE INSENSITIVE 3 (EIN3) and ETHYLENE INSENSITIVE 3-LIKE 1 (EIL1; Chao et al., 1997). For EIN3, the regulation appears to be direct since EIN3 can specifically bind the *ICS1* promoter (Chen et al., 2009). In addition, NPR1 has been reported to negatively regulate *ICS1* expression via an as yet unknown mechanism (Wildermuth et al., 2001; Zhang et al., 2010a). Up to now no pathogen effectors have been identified that directly target any of the transcriptional regulators for *ICS1* expression. We consider this likely to reflect the highly complex mode of regulation which may make *ICS1* regulation a much less attractive target for effectors than targeting SA accumulation or shifting the balance from SA to JA signaling (see below).

## Effectors Interfering with SA-Dependent Signaling and Gene Regulation

**FIGURE 2:**
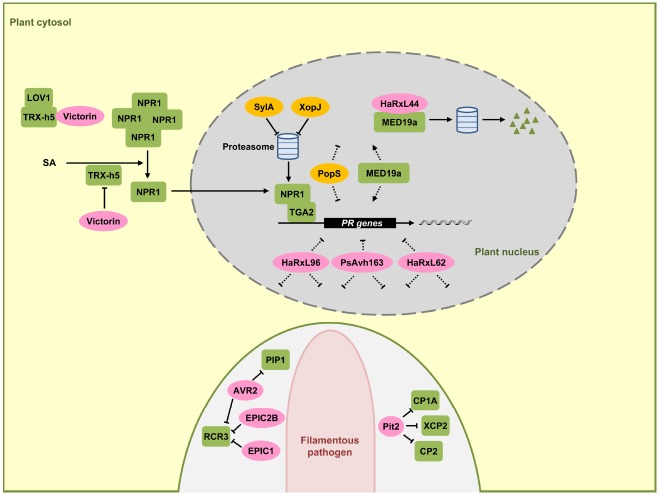
**Effectors interfering with SA-dependent signaling and gene regulation.** In this signaling scheme, we restrict the presentation to plant components targeted by virulence effectors. NPR1 is a central regulator in SA-dependent signaling pathway, triggering the expression of pathogenesis-related (*PR*) genes in the nucleus (gray) together with the TGA2 transcription factor. SA induces monomerization of cytosolic NPR1 oligomers with the help of thioredoxin TRX-h5. The mycotoxin effector victorin of *C. victoriae* can inhibit NPR1 through binding to TRX-h5 without causing disease. However, in LOV1 expressing plants victorin activates LOV1 with the consequence of cell death which is prerequisite for disease by this necrotrophic pathogen. *P. syringae* effector SylA and *X. campestris* effector XopJ act as proteasome inhibitors to suppress turnover of NPR1 and interrupt SA dependent defenses. HaRxL44 interacts with MED19a, leading to proteasomal degradation of MED19a. Oomycete RxLR effectors HaRxL96, PsAvh163, and HaRxL62, as well as a bacterial effector PopS, inhibit the expression of SA marker gene *PR-1* most likely indirectly. In the lower part a pathogen hypha is indicated in light pink. This hypha is surrounded by the apoplast shown enlarged here (light gray) encased by the plant plasma membrane (green). Effectors AVR2, EPIC1, EPIC2B, and Pit2 are secreted to the apoplastic space where they inhibit plant proteases PIP1, RCR3, CP1A, CP2, and XCP2 all induced by SA. Fungal and oomycete effectors are indicated by pink ovals and bacterial effectors are indicated in dark yellow. Plant components are indicated by green squares. Solid arrows represent direct activation, dotted arrows indicate indirect activation and blunt ended arrows indicate inhibition (for details, see text).

NPR1 is the central regulator of the SA signaling pathway and functions as a co-activator for an estimated 95% of the SA-responsive genes. When mutated, SA-dependent transcriptional responses are largely abolished and the corresponding mutants exhibit increased susceptibility to biotrophic and hemibiotrophic pathogens (Aravind and Koonin, 1999; Pajerowska-Mukhtar et al., 2013). In unchallenged plant cells, NPR1 resides largely in the cytosol in an oligomeric state that is stabilized by intermolecular disulfide bonds. Increases in SA levels after pathogen infection alter the cellular redox state (Mou et al., 2003), triggering a reduction of NPR1 by thioredoxins that leads to the dissociation of NPR1 into monomers (Tada et al., 2008). NPR1 monomers are then translocated into the nucleus where they interact with TGA-bZIP transcription factors (Zhang et al., 1999; Zhou et al., 2000), leading to an activation of SA-dependent gene expression including *PR-1* (Fan and Dong, 2002). TGA2 (Figure 2), TGA5, and TGA6 are transcriptional repressors of the *PR-1* promoter in the absence of SA and their repressive property may require interaction with additional components like NIMIN1, TOPLESS, and the CBNAC-SNI1 complex (Seyfferth and Tsuda, 2014). Once the transcriptional co-activator NPR1 resides in the nucleus, these previously repressing factors become positive regulators of SA-induced genes (Dong, 2004). NPR1 and related family members NPR3 and NPR4 bind SA and have been proposed to be SA receptors (Fu et al., 2012; Wu et al., 2012). While the true nature of the SA receptor is still debated (Boatwright and Pajerowska-Mukhtar, 2013), it is clear that nuclear localization of NPR1 is crucial for SA-mediated gene expression (Mou et al., 2003). To elicit an appropriate immune function, NPR1 activity in the nucleus needs to be tightly regulated. Nuclear NPR1 has been shown to be continuously degraded via the proteasome system in naïve cells to prevent untimely activation of immune responses (Spoel et al., 2009). SA stimulation has been shown to trigger phosphorylation of a phosphodegron motif in NPR1 facilitating NPR1 turnover. Phosphorylation-dependent turnover seems to be required for full activation of target gene expression, presumably indicating that NPR1 at the promoter needs to be replaced continuously to maintain gene induction (Spoel et al., 2009).

Although effectors directly targeting NPR1 have not yet been found in plant colonizing microbes, there are examples that some bacterial pathogens may indirectly target NPR1. The toxin syringolin A (SylA) from *P. syringae* pv. *syringae* inhibits proteasome function and the type III effector XopJ from *Xanthomonas campestris* pv. *vesicatoria* interacts with RPT6, a subunit of the proteasome complex crucial for proteasome function. The proposed model is that SlyA and XopJ may negatively influence the proteasome-mediated turnover of NPR1 to compromise SA signaling (Figure 2; Schellenberg et al., 2010; Misas-Villamil et al., 2013; Üstün et al., 2013).

The fungal pathogen *Cochliobolus victoriae*, the causal pathogen of Victoria blight disease on oat, also seems to indirectly target NPR1. *C. victoriae* secretes the mycotoxin effector victorin, an effector evoking defense. In this necrotrophic pathogen defense activation is prerequisite for virulence. Victorin binds to the active site of TRX-h5 (Thioredoxin-h5) inhibiting its activity (Lorang et al., 2012). TRX-h5 has been proposed to act as guard of LOV1, an NB-LRR protein. Production of victorin by the pathogen leads to LOV1 activation (Figure 2), resulting in a resistance-like cell death response which promotes disease (Lorang et al., 2012). In plants lacking *LOV1*, victorin treatment leads to reduced *PR-1* expression to levels comparable to *TRX-h5* mutants. This reflects the victorin-induced inhibition of TRX-h5 activity and lack of NPR1 monomerization. As such, victorin canonically functions as a virulence effector molecule in plants lacking *LOV1* by targeting thioredoxin (Lorang et al., 2012).

HopM1 and AvrE are representatives of conserved bacterial effector families which have in common the ability to suppress SA-dependent basal immunity and disease necrosis (DebRoy et al., 2004). The biotrophic bacterial wilt pathogen of tomato, *Ralstonia solanacearum* has the type-III effector PopS which is also a member of the AvrE family. This effector suppresses SA-mediated defense responses but fails to induce cell death (Jacobs et al., 2013). The targets for HopM1 and AvrE-type effectors with respect to SA signaling remain to be discovered.

The oomycete *Hyaloperonospora arabidopsidis* causing downy mildew in *A. thaliana* and *P. sojae* produce the effector proteins HaRxL62, HaRxL96, and PsAvh163, respectively, which are secreted proteins containing a N-terminal RxLR motif that is widely conserved in oomycete effector proteins that are delivered into host cells (Whisson et al., 2007; Anderson et al., 2012; Asai et al., 2014). HaRxL62, HaRxL96, and PsAvh163 effectors, all reduce transcription of the SA marker gene *PR-1* in transgenic plants when these are infected by an avirulent *H. arabidopsidis* strain or treated with SA (Anderson et al., 2012; Asai et al., 2014), suggesting interference with SA signaling. However, it is unclear which component SA-dependent plant defense response is suppressed by these effectors (Figure 2).

The nuclear-localized RxLR effector HaRxL44 of *H. arabidopsidis* interacts with the Mediator subunit 19a (MED19a), a positive regulator of plant immunity in *A. thaliana* (Caillaud et al., 2013). Mediator is a highly conserved multi-subunit complex that functions like a molecular bridge to facilitate the interaction between transcription factors at gene enhancer element sequences and RNA polymerase II at transcription initiation sites (Conaway and Conaway, 2011). The interaction of HaRxL44 with MED19a has been shown to induce the destabilization of MED19a by proteasome-dependent degradation (Figure 2). Transgenic plants of *A. thaliana* expressing HaRxL44 or *med19a* mutants show weak SA-triggered immunity and strong JA/ET signaling, illustrating that the degradation of MED19a shifts the balance from SA-responsive defense to JA/ET responsive defense, which is typical for many biotrophic pathogens (Caillaud et al., 2013). In addition to MED19a, Mediator subunits MED15 and MED16 are also shown to be required for SA-mediated resistance (Canet et al., 2012; Zhang et al., 2012).

Apoplastic proteases constitute a major component in plant defense responses. Benzothiadiazole, a functional analog of SA, facilitates accumulation of active papain-like cysteine proteases including PIP1 and RCR3 in the apoplast of tomato plants (Shabab et al., 2008). *Cladosporium fulvum*, the leaf mold pathogen of tomato, secretes the virulence effector protein AVR2. AVR2 adopts a highly compact structure through disulfide bonds involving its eight cysteine residues. AVR2 inhibits the cysteine protease activity of PIP1 and RCR3 by direct binding (Figure 2; Rooney et al., 2005; van Esse et al., 2008; van’t Klooster et al., 2011). *P. infestans* secretes EPIC1 and EPIC2B effector proteins that also act as protease inhibitors targeting tomato cysteine protease RCR3 (Figure 2; Tian et al., 2007; Song et al., 2009). Consistent with a role in defense, a tomato mutant in RCR3 exhibits enhanced susceptibility to *P. infestans* (Song et al., 2009). This illustrates that the defense-associated cysteine protease RCR3 is targeted by effectors from two unrelated filamentous pathogens (Figure 2). In maize, papain-like cysteine proteases also constitute a central component of apoplastic plant defenses. SA treatment of maize leaves strongly induces cysteine protease accumulation in the apoplast. SA-induced apoplastic cysteine proteases and their activity are sufficient to induce *PR-1* gene expression and the activation of plant defenses (van der Linde et al., 2012). Upon infection by *U. maydis*, maize cystatin CC9, a potent inhibitor of maize apoplastic cysteine proteases, is induced. Silencing of the *CC9* gene greatly attenuates *U. maydis* virulence (van der Linde et al., 2012), showing the importance of SA-induced cysteine proteases in maize defense signaling. In addition, the apoplastic virulence effector Pit2 of *U. maydis* (Doehlemann et al., 2011) interacts with and inhibits apoplastic maize cysteine proteases CP1A, CP2, and XCP2 (Mueller et al., 2013). This inhibitory activity depends on a novel 14 amino acid motif in Pit2. This motif is conserved in Pit2 orthologs of related smut fungi but does not exist in AVR2 or cystatins, which also inhibit members of the cysteine protease family (Mueller et al., 2013). SA-induced cysteine proteases are thus emerging as common virulence targets of filamentous pathogens (Figure 2). The need to inhibit this class of proteases by pathogen effectors may reflect that these plant proteases target core effectors important for virulence. Alternatively, these proteases could attack critical surface components of the pathogens. Current research aims to identify the important targets of these proteases.

## Effectors Targeting the Crosstalk between SA and JA Pathways

**FIGURE 3:**
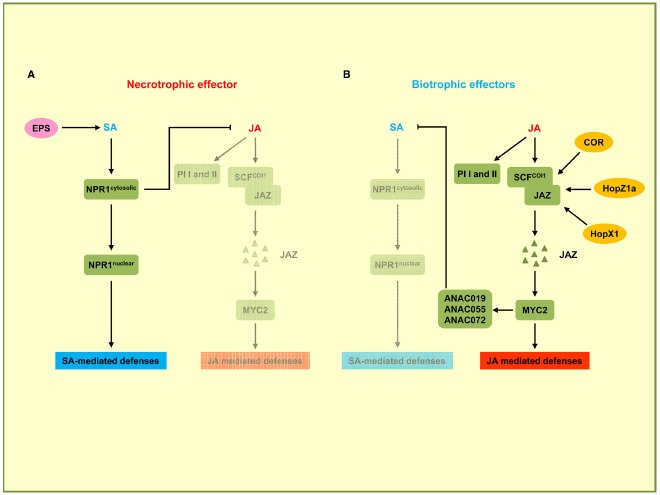
**Effectors targeting the crosstalk between SA and JA pathways.** In this signaling scheme, we restrict the presentation to plant components targeted by virulence effectors. **(A)** Necrotrophic effector activates the SA pathway while downregulating the JA pathway. *B. cinerea* uses exopolysaccharide EPS (depicted in pink) to activate SA-mediated defenses through NPR1 and to inhibit JA-mediated defenses including the expression of *PI I* and *PI II*. **(B)** Biotrophic effectors activate the JA pathway and suppress the SA pathway. *P. syringae* secretes phytotoxin coronatine (COR) to promote SCF^COI1^ ubiquitin ligase-dependent degradation of JAZ proteins. JAZ degradation activates MYC2, the transcriptional regulator of JA-responsive genes. MYC2 also induces NAC transcription factors ANAC019, ANAC055, and ANAC072 which are repressors of SA production. *P. syringae* uses HopZ1a and HopX1 to directly target JAZ proteins to accelerate their degradation, thus inhibiting SA-mediated defenses. Bacterial effectors are indicated in dark yellow. Plant components are indicated by green squares. Solid arrows represent direct activation, dotted arrows indicate indirect activation, and blunt ended arrows indicate inhibition.

There is extensive antagonistic crosstalk between SA and JA pathways which is exploited by pathogens to meet their specific needs (Gimenez-Ibanez and Solano, 2013; Kazan and Lyons, 2014). In the negative crosstalk between SA and JA, the activation of the SA pathway can confer susceptibility to plants upon the attack of pathogens that are restricted by the JA-dependent pathway, and conversely the activation of the JA pathway can suppress the SA pathway in favor of biotrophic pathogens (Figure 3). For instance, it has been shown that the NahG plants of *A. thaliana*, which are unable to accumulate SA, show 25-fold higher levels of JA and express JA-responsive genes (Spoel et al., 2003). In addition, several plant proteins regulating the SA–JA crosstalk have already been identified. *npr1* mutants, which are unable to respond to SA, show increased levels of JA and enhanced JA-responsive gene expression, indicating that NPR1 suppresses JA signaling (Spoel et al., 2003). Nuclear localization of NPR1 is not required for the suppression of JA-responsive gene expression, suggesting that cytosolic NPR1 may modulate the crosstalk between SA and JA (Figure 3; Spoel et al., 2003). In the JA signaling pathway, JAZ proteins, which are negative regulators for JA-responsive gene expression, are degraded by the E3 ubiquitin ligase SCF^COI1^ complex in response to JA. Subsequently MYC2, the transcriptional regulator of JA-responsive genes is activated. The *MYC2* gene is also required for the repression of SA-mediated defense responses (Figure 3; Laurie-Berry et al., 2006). *P. syringae* uses the phytotoxin coronatine (COR), a structural mimic of JA-Ile (the active form of JA in *A. thaliana*), for binding to the JA co-receptor COI1 (Xin and He, 2013). The COR-bound COI1 receptor complex promotes the degradation of JAZ proteins that act as negative regulators of the JA pathway (Figure 3). This leads to the activation of JA-responsive genes via MYC2, which also induces the transcription of three homologous NAC family transcription factor genes: *ANAC019*, *ANAC055*, and *ANAC072* (Zheng et al., 2012). These three NAC transcription factors repress the *ICS1* gene leading to a downregulation of SA production and signaling. In this way COR promotes susceptibility to *P. syringae* by suppressing SA signaling (Brooks et al., 2005). The function of COR to induce JA responses can also be carried out by bacterial effector proteins. HopZ1a, an effector of *P. syringae*, directly acetylates JAZ proteins. This leads to COI1-dependent degradation of JAZ proteins (Figure 3), resulting in an induction of JA-mediated defenses and a concomitant repression of SA responses (Jiang et al., 2013). The JAZ proteins are also targets of HopX1, another *P. syringae* effector encoding a cysteine protease that interacts with and promotes the degradation of JAZ proteins (Figure 3; Gimenez-Ibanez et al., 2014).

The necrotrophic fungal pathogen *Botrytis cinerea* secretes a non-proteinaceous exopolysaccharide (EPS) effector, β-(1,3)(1,6)-D-glucan (El Oirdi et al., 2011). EPS from *B. cinerea* exploits the antagonism between the SA and JA pathways to promote fungal virulence. Tomato plants pre-treated with the EPS show significantly elevated SA levels and disease susceptibility, and conversely a reduction of JA-dependent defense genes *PI I* and *PI II*. *PI I* and *PI II* code for proteinase inhibitors required for resistance against *B. cinerea*. When EPS is applied to *NPR1*-silenced plants, increased SA accumulation and disease susceptibility are not observed, indicating that EPS-induced disease susceptibility is likely to occur through NPR1. These results demonstrate that *B. cinerea* EPS activates the SA pathway through NPR1 for promoting disease and concomitantly represses the JA pathway that would restrict virulence of this necrotrophic pathogen (El Oirdi et al., 2011).

## Conclusions and Outlook

While it is becoming increasingly clear that all biotrophic pathogens (as well as hemibiotrophs during their biotrophic phase) need to suppress SA signaling to cause disease the molecular details of how this is achieved by effectors in the various systems is only beginning to be understood. Given the small number of examples where pathogen effectors targets in these processes have been identified, it is probably not surprising to see little if any overlap between prokaryotic and eukaryotic virulence effector targets. This picture is very likely to change once more effector targets are discovered.

Is there an advantage of interference at the level of SA biosynthesis, SA signal transduction and gene regulation or the antagonistic interplay between SA and JA signaling over interfering with PAMP perception directly at the level of the receptor (Macho and Zipfel, 2015)? We think so, because targeting the activity of a certain PRR would be highly specific while interference with SA signaling further downstream affects the response at a level where signaling pathways have converged. Also, in view of the fact that plants are estimated to have hundreds of PRRs with ligands presently mostly unknown, effector interference at a more downstream level could provide a common response to different PAMP triggers. In addition, effector interference at the level of the PRR might not appropriately allow accommodation of the different life styles of pathogens, i.e., necrotrophs that activate SA signaling, biotrophs that activate JA signaling or hemibiotrophs that switch from one to the other mode of signaling. Thus, maintaining this flexibility may be a key to pathogen success. This is also likely the reason why certain pathogens have developed several effectors interfering with the same pathway, albeit at different levels. Given the more than 10-fold greater abundance of effectors in eukaryotic pathogens compared to bacterial pathogens, we also wonder whether redundancy will suffice as an explanation. In the *U. maydis*-maize system effectors are deployed in an organ-specific manner (Skibbe et al., 2010) explaining different needs for effectors in discrete organs. In addition, eukaryotic pathogens undergo a series of infection-related developmental processes in the infected tissue, which may require a reprogramming of the host in specific ways, conceivably involving alternative effectors. These considerations show that current work on effectors is just scratching the tip of the iceberg, and a lot of exciting science is still to come.

### Conflict of Interest Statement

The authors declare that the research was conducted in the absence of any commercial or financial relationships that could be construed as a potential conflict of interest.
